# People Centeredness, Chronic Conditions and Diversity Sensitive eHealth: Exploring Emancipation of the ‘Health Care System’ and the ‘Patient’ in Health Informatics

**DOI:** 10.3390/life10120329

**Published:** 2020-12-07

**Authors:** Lars Botin, Pernille S. Bertelsen, Lars Kayser, Paul Turner, Sidsel Villumsen, Christian Nøhr

**Affiliations:** 1 Danish Centre for Health Informatics, Techno-Anthropology & Participation, Aalborg University, DK-9000 Aalborg, Denmark; pernille@plan.aau.dk; 2 Health Informatics & Innovation, University of Copenhagen, DK-1353 Copenhagen, Denmark; lk@sund.ku.dk; 3 eHealth Services Research Group (eHSRG), University of Tasmania, Tasmania, TAS 7001, Australia; paul.turner@utas.edu.au; 4 Human Resource Development, Central Denmark Region, DK-8200 Aarhus, Denmark; sidsel.villumsen@stab.rm.dk; 5 Maersk Mc-Kinney Møller Institute, University of Southern Denmark, DK-5230 Odense, Denmark; cn@mmmi.sdu.dk

**Keywords:** eHealth, end-user, chronic disease, participatory design, socio-technical, diversity in care

## Abstract

Health care systems struggle to consistently deliver integrated high-quality, safe, and patient-centered care to all in an economically sustainable manner. Inequity of access to health care services and variation in diagnostic and treatment outcomes are common. Further, as health care systems become ever more complex, iatrogenesis and counter productivity have emerged as real dangers. In exploring this paradox, this paper considers a subset of those in society living with chronic conditions. Their attributes and circumstances have led to them being marginalized or excluded from ‘end-user’ engagement and/or from their requirements being incorporated into technology supported chronic disease management initiatives. Significantly, these citizens are often the most vulnerable and socially disadvantaged and tend to achieve poorer results and cost more per capita than the ‘average patient’ in their interactions with the health care system. Critically, this paper argues that a truly people-centered technology supported chronic care system can only be designed by understanding and responding to the needs, attributes and capabilities of the most vulnerable in society. This paper suggests innovative ways of supporting interactions with these ‘end-users’ and highlights how reflection on these approaches can contribute to emancipating the health system to move towards more socially inclusive eHealth solutions.

## 1. Introduction

Chronic diseases (multimorbidity or complex chronic conditions) are a major health care burden on the health care systems of most developed countries [1]. Numerous efforts and approaches have been advocated to address the challenges associated with the burden of chronic disease [2]. In particular, one group of approaches [3,4,5,6] have advocated an integrated approach to care delivery and management that directly involves the patient as a co-participant in their own care and promotes the idea of empowering the patient to achieve higher levels of self-efficacy for self-management to support better health outcomes (i.e., enhanced self-care, awareness, capacity to access/benefit from interactions with service providers, improved health outcomes).

More recently, many of these approaches have been supported by the deployment of technologies with the aim of enhancing information sharing and communication [7]. To date, the results of the implementation have met with mixed success partly due to:

- Implementation challenges arising due to fragmentation of existing health care delivery systems [8];
- A tendency to not use ‘state of the art’ technology;
- A tendency to interact with patients primarily through the lens of their illness rather than more holistically [9].

More significantly, it is also evident that these approaches presume a level of health and eHealth literacy on the part of citizens as a pre-requisite for their meaningful participation in these care delivery approaches. In this context, it has become apparent that there are large numbers of citizens who, as a result of a range of circumstances and personal attributes, are either not engaging with these approaches or when they do, tangible benefits are more difficult to identify due to their contexts [10]. This is why we call for a ‘diversity sensitive’ approach that is capable of seeing and hearing the most vulnerable and disadvantaged in designing solutions that takes this user diversity seriously. Furthermore, the design of new eHealth services and mechanisms for engaging ‘end-users’ in chronic disease management and self-management continues to unintentionally re-enforce these inequalities.

We start by documenting how it is mutually agreed that involving people with chronic diseases in their own health care is the way forward to meet the current Western health care challenges, both to minimize cost and to improve health.

Then we challenge this mutual agreement by requesting a need for a more diversified perspective on citizens with chronic diseases. We claim that by taking a point of departure in an archetype of disempowered, disadvantaged, and disconnected citizens with chronic diseases, there is achievements to be made by not only this group of citizens’ compliance to health care but also to the general compliance and development of assistive technology.

Further, we suggest three dimensions of marginalization that citizens with chronic disease can be challenged by, and introduce two models for (a) how to engage, empower, and emancipate the challenged groups of citizens, and (b) how to understand and classify use and non-use.

We then argue that focus on this group of citizens has the potential to re-invigorate and emancipate health systemic dysfunctions. The we introduce the 7 E of Techno-Anthropology grouped in two as a conceptualization of a critical ecology of technological systems and advocate for a participatory and socio-technical understanding of the interrelationship between people, health, and technology. Finally, we introduce a framework for inquiry with an outset in eHealth and point at three concrete approaches—giving, walking, and pushing—that can direct actions in appropriate ways.

## 2. Disadvantage, Chronic Disease, and Patient Engagement

Since Ed Wagner in 1995–1996 introduced the Chronic Illness Care Model (CICM) [3], later termed the Chronic Care Model (CCM), based on twenty years evidence on the importance of involving people in their own condition and to redesign the delivery of chronic care, several models have been developed [11,12,13,14,15]. More recently, at the political level in the European Union, it has also been realized that redesign of health care is essential to address the challenge of inequity of access and inequality in health outcomes [16]. National strategies address patient participation as a goal [17].

Unfortunately, most of these approaches aim to address differentials in health service provision from a systems perspective. This has led to a ‘system myopia and/or blindness’ because even where these approaches pay lip service to the increasing importance of the health literacy level of people and the need to involve patients in their own decisions, in all cases, they rely on the requirement that people are visible and are compliant with the way the communication is organized [18].

Significantly, evidence highlights that people with low health literacy are known to adhere less to health-promotion programs such as screening and vaccinations, have a higher morbidity, more visits to emergency rooms, and earlier mortality [19]. Even where the health care system interacts with these citizens and/or makes a formal diagnosis of their conditions, failure to understand what motivates and drives their habits and preferences in everyday life and how their preferences are influenced by their social relations and contexts results in continued poor health outcomes for these groups of patients [20]. To address this challenge, it is essential that new services such as matrons, patrons, and health navigators are designed and tailored to accommodate the needs and contexts of these most vulnerable patient groups. However, who are these individuals? Further, how do they currently interact with the health care system?

Data from a National Danish citizen survey from 2017 show how people with only primary school education are less likely to use already developed eHealth services. The eHealth services, whether private or public, are still emerging, but data show a clear tendency. The Danish national health portal Sundhed.dk was used less by those with low education, 21% with only primary school education and 60% of people with a high education [21]. For applications (Apps) developed for health purposes, citizens with only primary school education have less experience than those with high education (8% vs. 26%). The study shows a significant difference between citizens with low and high educational level, when it comes to opinion on, knowledge of, and experience with the use of eHealth and other internet, apps, or mobile services [21].

Showell and Turner (2013) described a deliberately simplistic binary distinction between ‘people like us’ (PLUs) and ‘Disempowered, Disengaged and/or Disconnected’ (DDDs) to stimulate consideration of how disadvantage contributed to the invisibility of certain types of user needs in the design of personal health record systems [22]. PLUs and DDDs, those who are more or less likely to adopt personal health records, were described as follows:

“Our high-uptake cluster includes people who understand health care and health issues, take care of their own health, are literate, well to do, tech-savvy, and hold a tertiary qualification. We recognised ourselves as being members of this group [PLUs]. Those within our low uptake cluster are disinclined to take exercise for its own (or their own) sake, or to eat sensibly. They are not text-, health- or technically-literate. They struggle financially and may not have finished secondary education [DDDs]. This simplistic characterization provided archetypes of two extremes and pointed to factors which could affect eHealth uptake” [22].

This is a useful and provocative distinction to draw attention to how contemporary eHealth mechanisms may be contributing to a widening of the health and eHealth divide. However, if we are to develop more targeted solutions, it is important to also recognize that DDDs are far from a homogenous group, and it is necessary to investigate and unpack this simplistic classification. In order to reflect and respect the fact that the group is heterogeneous and multiple on all levels, might that be socially, culturally, and economically, then we have opted for a need for a ‘diversity sensitive’ approach, which hopefully will be able to look and hear beyond the evident and simplistic definition of both the DDDs and other citizens. Another approach that has recently dealt with the engagement and involvement of the vunerable and challenged is characterized by focusing on the clinical frailty of the impaired [23,24]. This frailty is multiple as it characterizes what goes on at professional, clinical, social, and cultural levels. In this paper, we have focused on the ‘diversity sensitive’ approach to DDDs, because we believe that the cognitive frailty of the impaired is part of marginalizations that take place.

## 3. Three Dimensions of Marginalization

For people with chronic conditions, there are already both numerous generic and disease-specific instruments that have been developed to determine an individual patient’s levels of self-efficacy and capacity for self-management aimed at supporting the tailoring of health services. For example, SF36v2 and HeIQ are regularly used to identify and track patient’s function and well-being in relation to health care interventions. Significantly, however, the utility of these instruments and the data they produce is intimately tied to individual patient’s commitment to participate and engage with the health care profession. To spend time with a care provider and fill in forms or to be interviewed, these instruments are first fully functional when people are convinced to interact with the health care system. The commitment is the first dimension of the marginalization of DDDs, who, if they live with chronic conditions, tend not to enroll or be recruited into initiatives developing new models of health service delivery.

The second dimension of marginalization arises unintentionally from pro-active efforts of many health system initiatives to educate and engage citizens to improve their capacities to more actively participate in care delivery processes. Again, these worthy efforts rarely engage with DDDs living with chronic conditions, who, for a variety of reasons including social disadvantage and low levels of textual, technical, and health literacy, either do not engage or acquire limited benefit from engagement because of the design, structure, and processes used in these initiatives. Unfortunately, this is partly evidenced by the fact that citizens who could be classified as DDDs do continue to have regular contact with the health care system, they do use system services usually for longer or more frequently than ‘average citizens’ yet they continue to achieve poorer results than many other more health literate and/or eHealth literate citizens.

The third dimension of DDD marginalization relates to critical reflection on the very notion of people centeredness. Most contemporary efforts to engage citizens involve bringing them closer to the existing health system by finding ways to empower citizens to fit the existing service offerings rather than tailoring service offerings to needs, capabilities, and contexts of citizens. Perhaps, unsurprisingly, one net effect of many of these initiatives has been to stimulate an increase in the use of health services by the ‘worried well’ and/or PLUs but this in turn has the potential to further inhibit recognition of the need to find DDDs in their own contexts and to interact with them in ways designed to be meaningful and effective for them.

## 4. Models for Identifying and Classifying DDDs

**Figure 1:**
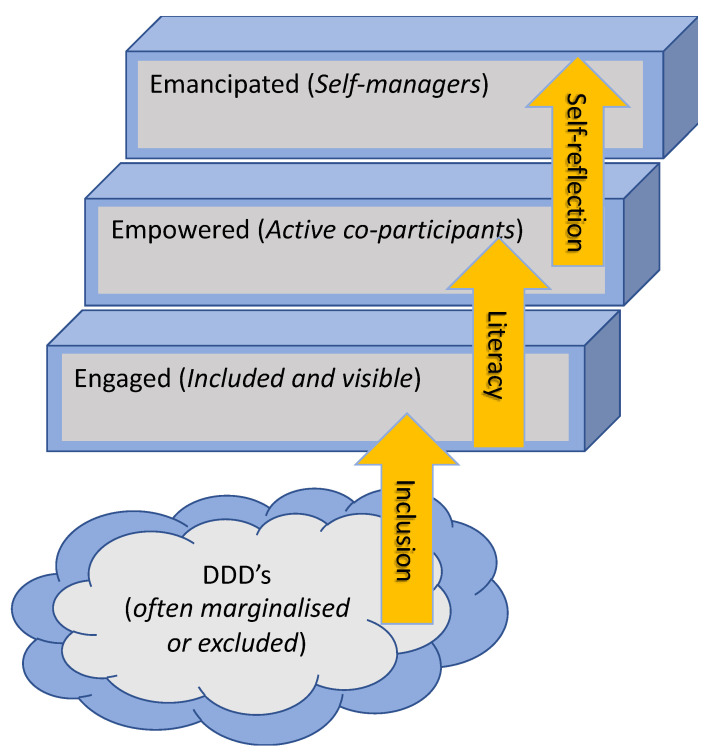
The Disempowered, Disengaged and/or Disconnected (DDDs) are often unintentionally marginalised or excluded from chronic disease management models.

Figure 1 below illustrates how people living with chronic conditions can be categorized as being at different levels in relation to their capacities and skills to engage with disease management models. People with a diagnosis in the health care system often have differentiated levels of knowledge, competences, and skills from being engaged through to being emancipated, where they can comfortably self-manage. Citizens in the *engaged phase* are encouraged to adhere/comply with service delivery and exhibit relatively lower levels of self-efficacy for self-management. In other words, they are highly reliant on the service delivery and have limited personal resources or knowledge of their own condition or of how and why the system works in the way it does. In order to move to the *empowered phase*, people need to become further engaged and supported with tools and methods for enhancing their position in relation to the system and in relation to their personal health condition. This is facilitated by them having or acquiring higher levels of health literacy and eHealth literacy, such that the patient is capable of making use of existing and new solutions for personal change and transformation of attitude and behavior in relation to the management of their chronic condition in collaboration with the health profession [25]. A concrete tool for supporting transition to this empowerment phase is the Behavior Change Support System (BCSS), described as “a socio-technical information system with psychological and behavioral outcomes designed to form, alter and reinforce attitudes, behaviors and/or acts of complying without using coercion or deception” [26]. This highlights that the important transition is that people do not experience coercion or deception as they are guided and empowered by the system and the various tools that are applied in order to change compliance, behavior, and/or attitude.

Transitioning to the *emancipation phase* involves self-reflection and in-depth knowledge of one’s own health condition, high levels of health and eHealth literacy, and detailed understanding of the how’s and the why’s of the system. True emancipation can only occur through communication/dialogue where there is no master or dominance performed [27], meaning that the system has to ‘step back’ in order for the citizen to realize and genuinely become emancipated. Jürgen Habermas also points at the fact that in order for this to happen, we have to abolish our focus on the individual and the subject, and create a platform for communicative intersubjectivity [27], where the needs and requirements of the weak and vulnerable are given the possibility to emerge and become visible in this communicative formation of intersubjective action and understanding. It is obvious that by this we do not mean to overcome the system of clinical expertise and leave it all to the individual being self-reflective and self-managing. The health care system should in this process experience the same emancipation from external and ‘hostile’ stakeholders and hence fully partake in the progress and development in chronic disease management models.

Figure 1 is inspired from [25] and aims to represent these different trajectories of engagement that different types of citizens experience through chronic disease management to highlight how DDDs experience significant personal and systemic barriers to benefiting from engagement with these initiatives.

In an attempt to unpack the concept of DDDs living with chronic conditions, it is important to firstly acknowledge that they are not just patients but also people. Some of these individuals are already interacting with the health care system but many others are not, even though they may be engaging in lifestyles that will eventually lead to a requirement for health system services.

**Figure 2:**
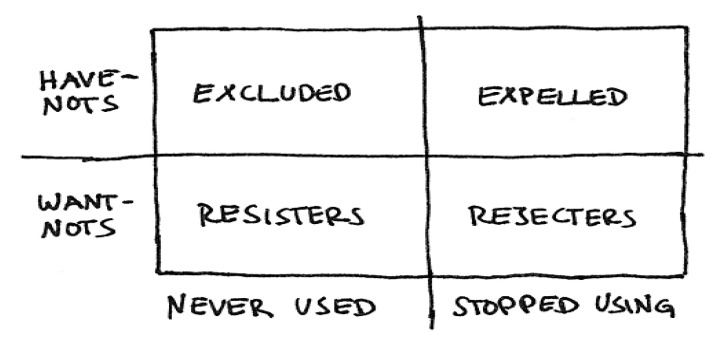
Wyatt’s classification of non-users of the Internet (2003) [28].

A useful way to conceptualize some of the differences amongst DDDs with chronic conditions (diagnosed or not) towards engaging with health/eHealth systems builds on socio-technical analysis conducted by Sally Wyatt about users and non-users of the Internet [28]. In examining non-users of the Internet, Wyatt identified two categories (‘Have nots and Want nots’) who could be further divided into groupings based on whether they never used the Internet (Resisters or Excluded) or had stopped using the Internet (Rejecters or Expelled), see Figure 2.

This classification opens up consideration of how different attributes amongst DDDs may influence their engagement with technology supported chronic disease management initiatives. It also illuminates the challenges around designing mechanisms to connect with these different types of non-users. It also usefully highlights the methodological insight that as well as identifying and engaging with end-users to understand their situational and contextual knowledge, skills, and interests, it is likely to be equally important to identify non-users as part of input into re-design of health and eHealth solutions for the chronically ill. A related methodological point is that when formulating responses to enhance DDD engagement, empowerment, and potentially emancipation it is necessary to acknowledge that these are themselves far from fixed or static concepts. As such, unlike more well-known measures (e.g., self-efficacy and self-determination), they are yet to be well grounded as concepts.

Having briefly considered DDDs living with chronic conditions as an archetype in need of new and innovative approaches to support their interaction to improve health outcomes, how should we conceptualize existing health systems and the different drivers that have contributed to its continued dysfunction in this regard?

## 5. Being and Acting in the Technological Eco System

Beyond the micro level of personal and social contexts, people living with chronic conditions experience meso- and macro-level structures of contemporary health systems that shape and mediate their interactions with these systems. The inherent hierarchies within contemporary health care systems are well known and have previously been investigated by writers including Michel Foucault (1963) and Ivan Illich (1975) [29,30]. Indeed, despite the prevalence and pervasiveness of the mantra of the need for the system to be ‘patient centred’, it is evident that most patients remain far from the locus of power, control, or self-determination and continue to be objectified as ‘bundles of symptoms’ rather than as subjective co-participants in their own treatment and care.

It is in this context that DDDs both as end users (marginalized by the system) and non-users (excluded from this system) have the potential to contribute directly to re-invigorating and emancipating health systemic dysfunction by making it visible and providing insight in how and why this system fails to enfranchise the most vulnerable in society living with chronic conditions. Critically, however, if the system is to be emancipated new mechanisms must be identified that can allow health and eHealth professionals to literally walk with DDDs and not just talk to them, i.e., to create a platform for just, fair, and equal communicative intersubjectivity. There is a need to find ways of capturing insights and experiences from DDDs living with chronic conditions and using this diversity sensitive knowledge as stimuli for re-designing structures and processes to enhance health outcomes for all those living with chronic conditions. This suggested approach is not mere fantasy but rather based on considerable evidence from within the ‘science and technology studies’ literature where there are numerous examples of technological developments and implementations being improved as a result of resistance and rejection including the car, the bicycle, and the telephone [28]. Enrico Coiera pointed at the same fact as he, in a very illustrative way, described how workarounds could be beneficial for the evolvement of appropriate technologies: “We should thus see workarounds as gifts. Rather than representing a problem with the way users engage with a technology, workarounds are clear signals that there is a mismatch between work as imagined, and work as done. Indeed, we can think of *workarounds as repair*, providing *missing information, new pathways or tools* to improve system’s fitness of purpose. -They are user’s attempts to fix inadequacy in design and to meet emergent or unanticipated needs ” [31]. Coira is describing how Wyatt’s *resisters* are creating models and ways for alternative and often more appropriate technological modes of work and interaction.

## 6. 7 Es of Techno-Anthropology for a Critical Ecology of Technological Systems

Following Susan Leigh Star (1999), there is a need to reflect on contemporary health system approaches to the management of chronic conditions as a ‘boundary object’. This enables a re-conceptualisation of the operation of power and influence and better supports finding ways to open up and re-negotiate how the system operates [32].

Following Bowker and Star (2000):

“If both people and information objects inhabit multiple contexts and a central goal of information systems is to transmit information across contexts, then a representation is a kind of pathway that includes everything populating these contexts” [33].

Bowker and Star provide a short list of requirements for an ‘ecological understanding’ to occur that includes understanding:

1. How objects can inhabit multiple contexts at once and have both local and shared meaning.
2. How people, who live in one community and draw their meanings from people and objects situated there, may communicate with those inhabiting another.
3. How relationships form between (1) and (2) above. How can we model the information ecology of people and things across multiple communities?
4. What range of solutions to these three questions is possible and what moral and political consequences attend each of them? [33].

This list raises a number of critical questions for what concerns the ‘ecology’ of any technological system including multiple contexts; local and shared meaning through communication between communities; and, moral and political consequences. The British anthropologist Edward Hall (1966) has coined this approach *proxemics*, meaning that we as facilitators or catalyzers of new and appropriate understandings of local communities have to be close/intimate (proximal) and acting within the social group (emic), because: “The emic approach investigates how local people think: How they perceive and categorize the world, their rules for behavior, what has meaning for them, and how they imagine and explain things” [34].

Furthering the discussion on how to reach an ‘ecological understanding’ of how to get closer and how to act in intersubjective communities, Lars Botin has introduced 7 Es in interventionist and value-based research and technology [35]. The 7 Es cover: engagement, empathy, embodiment, enactment, enhancement, empowerment, and emancipation. The first four Es are concerned with the specific approach that accordingly is required in order to get close and to act within the social group. We have to be there physically and embrace with empathy in order to enact change. The last three Es are concerned with the aims for our intervention. Why do we engage and enact together with the DDDs? We do that in order to enhance, empower, and emancipatethe system(s), the experts, the DDDs, and ourselves as an integrated part of this process.

Considering these questions in relation to DDDs as a community that ought to be engaged, empowered, and emancipated by the system, and not excluded or marginalized, as is currently the situation, is useful. The DDDs then, in line with the thoughts of Enrico Coira, emerge as having the potential as change agents for improving health system management of chronic conditions.

In this context, one argument presented here is that there is the possibility of a positive dialectic such that DDDs can be better engaged, empowered, and potentially even emancipated by change simultaneously as the health system is emancipated from its contemporary dysfunction. Emancipation of the health system is not an utopian ideal but rather has to be considered as involving both structural process and individual re-design, based on the experiences of DDDs.

## 7. Engagement, Empowerment, and Emancipation: New Approaches with DDDs

“If One is Truly to Succeed in Leading a Person to a Specific Place, One must First and Foremost Take Care to Find Him Where He is and Begin There (…) all true helping begins with a humbling. The helper must first humble himself under the person he wants to help and thereby understand that to help is not to dominate but to serve, that to help is not to be the most dominating but the most patient, that to help is a willingness for the time being to put up with being in the wrong and not understanding what the other understands” [36].

The Danish philosopher Søren Kierkegaard stated that if you want to help another person, you will need to start where that person is [36] or as Nelle Morton [37] told us: ‘listen in order to make people speak’. The German philosopher Martin Heidegger pointed at the importance of choosing different strategies and tools in order get closer to a better and trustworthy picture of reality: “A strange measure (…) certainly not a palpable stick or rod but in truth simpler to handle than they, provided our hands do not abruptly grasp but are guided by gestures befitting the measure here to be taken. This is done by a taking which at no time clutches the standard but rather takes it in a concentrated perception, a gathered taking-in that remains a listening” [38]. We are told to gently and carefully approach the world and listen. This is a completely different way of dealing with reality that transcends scientific measurement and calculation, and it makes way for different voices to be heard and also hearing the diversity of these voices.

This paper argues that a truly people-centered technology supported chronic care system can only be designed by understanding and responding to the needs, attributes, and capabilities of the most vulnerable in society—the DDDs.

**Figure 3:**
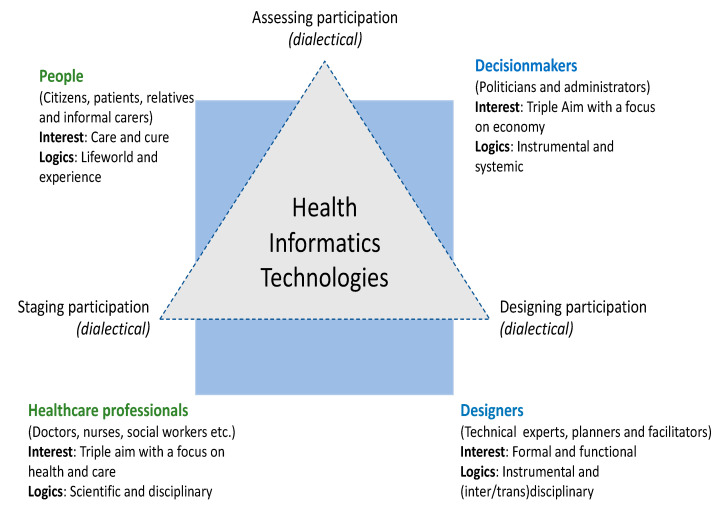
Techno-Anthropological frame for inquiry with an outset in eHealth.

In the figure below (Figure 3), we have attempted to frame how participatory inquiry and design of inclusive technology can be made, considering all the potentially involved parties, i.e., people (DDDs), health care professionals, decision makers, and system designers. This is made within the socio-technical framework of Techno-Anthropology, which is an interdisciplinary, participatory, and value-based approach to sustainable and responsible technological innovation, development, implementation, and use [39].

In the approach, there is a focus on how to meet and challenge the dominant technological and systemic rationale and logics of modern Western society, which manifest on the individual, institutional, and societal scales. In the figure, we address how staging, designing, and assessing participation happens in between identified groups and communities, and furthermore, how this evolves in relation to technologies. We find that this process is dialectical on multiple levels and in relation to a multitude of users and non-users, where the focus of this paper is on how to engage, empower, and emancipate people in need of health care and eHealth. We believe that the interests and logics of people, i.e., citizens, relatives, and informal carers, are characterized by a need and wish to receive and produce care in order to cure from a current state of health. These needs and wishes are interdependent with their current lifeworld conditions and experiences, which are again multiple and different, dependent on who and what you are.

Figure 3 also shows how other groups/communities are present and activated in the process, and how these are implicitly interconnected. Health care professionals are connected to people through technologies that mediate cure and care, and at the same time they have interests and logics that are in touch with both the decision makers and designers, i.e., they think and act professionally and as experts with a high degree of scientific disciplinarity/rigor, instrumentality and structural systemic procedure. We notice a clash in between interests and logics, where health care professionals are often caught as hostages in between the systemic and political rationale of efficiency and economic optimization, and a sincere wish to alleviate and improve the fragile and vulnerable condition of exposed and excluded citizens, i.e., DDDs.

We have placed technologies in the center of the figure, because we are of the opinion that technologies could/should mediate a transition from a technical and systemic focus on efficiency and optimization, to an interactive concern on how to create platforms (technological) for intersubjective communication on engagement, empowerment, and emancipation. On the right-hand side of the figure we have placed the ‘decisionmakers’ and ‘system designers’, roughly indicating their logics and interests as instrumental and systemic/disciplinary, which is of course a very mechanical and squared categorization. Many health care professionals are, as we were saying, institutional part of the decision-making process and care for the standards of ethical and emphatic medical practices that should be ‘inscribed’ in technological systems. Designers are not isolated in their technical design-processes but ‘listen’ to planners and facilitators, whom might be health care professionals with a particular interest or competence in health informatics. Many health information systems have been developed through and with the engagement of health care professionals. Nevertheless, it is the assumption that health care professionals that engage in the invention, development, and implementation of health information systems are not represented in Wyatt’s figure (Figure 2), in relation to the expelled, excluded, rejected, and resisters of technological systems. This means that many of the voices of the marginalized or those moving on the borders of the systems, and possibly challenging and confronting the technological innovation, are not heard or considered in potential interdisciplinary and transdisciplinary collaborations. Techno-Anthropology would ask the critical question of how to challenge and confront technological innovation from the inside out, which means that we are not looking (critically) from the outside, but rather trying to get inside and from the border looking out and in at one and the same time. The system has to be open for ‘critical proximity’ (Latour) to be performed, hence allowing challenging and critical ‘voices’ to penetrate the membrane of the system.

## 8. Speculative Approaches to Diversity Sensitive Attention

Kirkegaard and Heidegger stressed the importance of attending the other with care and cure, and this could be done by listening. There are complementary ways of relation to the other, which will be described in the following. We are fully aware of the fact that these complementary approaches, which are characterized by a higher degree of *action* than the humble listening to the other, are insufficient to grasp the kaleidoscopic reality of diversity and sensitivity. On the other hand, we are convinced that *giving, walking, and pushing* requires a high degree of attention and reflections on intentions that will direct our actions in appropriate ways.

### 8.1. The Gift

The French anthropologist Marcel Mauss pointed at the fact that there is a double edge and meaning in the *gift*. It is both a giving and a sharing of a thing, and a possible poisoning of relationships [40]. Hence, we should be careful in our giving, and reflect upon the inherent asymmetry between giver and receiver of the gift in order to prevent the spreading of poisonous feelings and emotions. What do we expect in return for our gift, and how can we make the receiver understand that the return does not have to be of equal value? Mauss paraphrases the American novelist and philosopher Ralph Waldo Emerson by saying: “charity wounds him who receives, and our whole moral effort is directed towards suppressing the unconscious harmful patronage of the rich almoner” [40]. So, it is a moral effort that has to be made by the giver as she handles over the gift to the vulnerable and marginalized individual, whom by receiving is put into a position of subjugation and submission. Mauss’ moral conclusion is that: “It is our good fortune that all is not yet couched in terms of purchase and sale. Things have values which are emotional as well as material; indeed in some cases the values are entirely emotional. Our morality is not solely commercial” [40].

Technological systems are hardly ever perceived as a gift, but rather as some sort of coercion or ‘enframing’ of procedures and behaviors of both citizens, relatives, informal caregivers, and health care professionals. However, the data delivered to the system are from patients who upload or hand over personal data (verbally, written, or monitored) that will be received by the health care professionals. The health care professionals act upon the data and confirm or correct the care plan. The question remains how to turn this perception into a more accommodating and positive stance.

### 8.2. The Walk

Kanstrup et al. (2014) points at ‘design with the feet’ in participatory design (PD) perspective [41]. We want to suggest this method, inspired by Transect Walking, as a way to engage citizens by listen, talk, and walk in their reality. Going for a walk is a way to open up a conversation. Not only in order to engage in specific health-related issues, but exactly in order to physically engage with people. Walking is a way to gain access to people’s knowledge. Here, the context can act as a common third that connects citizens and a visitor to the conversation and contributes to opening up for insights into, e.g., difficult issues in the citizen’s life. The focus is not on each other’s faces, but the scenery both see. This approach allows starting both difficult and problematic conversations.

The British novelist and anthropologist Bruce Chatwin walked the paths of many marginalized people in the world, and among them the aboriginals of Australia. In the book *The Songlines* (1987), he describes how the only way to understand the people (the aboriginals) is to follow their paths in the dreamland of the land [42]. There are different songlines that, dependent on your person, experiences, competences, and skills, will direct you as you walk the land. We, as participants in the journey, can learn from the songlines and get in-depth knowledge about the traveler, hence open up for inclusion. In a diversity sensitive approach, we should be seeing and listening to the qualities of their journey and we should learn from the stories told on this journey.

On this note, the importance of the *other* becomes paramount, and how we interact with the *other*, is crucial when it comes to co-creation and co-constitution of meaningful platforms (technologies) for engagement, empowerment, and emancipation. Kirkegaard pointed at this, as he recommended a humble and serving attitude towards the other in need of guidance and help. Hannah Arendt (1958) had a strict focus on how good life is constituted through togetherness, and that it is only through our interactions with others that we recognize and acknowledge who we are [43]. Recent philosophy of technology has pointed at the fact that technology is co-constitutional in these processes [44,45,46,47], and that technology should be seen as a co-determining factor when it comes to intersubjective, interrelational, and interactive attempts toward the creation of good and meaningful life.

### 8.3. The Push

Pushing must be emphatic and enacting in order to escape coercion and/or deception. Secondly, pushing has to be experienced (embodied) as something that happens for the better of personal situation and condition. Through pushing (or nudging) participants have to experience the opening of the system in relation to personal needs, wishes, and requirements. That means that pushing has to be an act of responsibility, where the system can be held accountable for actions: equity, fairness, justice, and stewardship. Steven Dorrenstijn and Peter-Paul Verbeek state that: “User-influencing design methods (nudging) can help to prolong the tradition of socially engaged design, with tempered, non-utopian goals, but at the same time with improved understanding and more effective tools concerning how technology mediates our existence” [48] (our brackets). According to Dorrenstijn and Verbeek technological solutions for pushing has to be ‘socially engaged’ and ‘tempered’, which means that when we stretch efforts to the limit in a flow of mutual enjoyment and fun, then these efforts should be *tempered* by and through technology. Thaler and Sunstein (2008), who introduced to the concept of nudging as a way of ‘improving decisions on health, wealth, and happiness’, met the dilemma of coercion versus autonomy with the possibility to opt out [49]. We, as citizens, users, consumers, patients, etc., should have the possibility of saying no, which of course can be considered as an escape way from undesired bonds, but on the other hand it gives the false impression of being liberated from these bonds, which are often technical. Dorrenstijn and Verbeek points in the opposite direction as they indicate opt in as a way of gaining a sense of freedom and well-being [48]. We want DDDs to opt in as they are called for engagement and subsequent empowerment and emancipation. Technology should in this way pave the path (mediate) for opting in and by this mediate a sense of freedom in practice, where it is not the case of saying yes or no, but rather of ‘accompaniment’ [50]. Technology as a companion in surmounting thresholds, and hence facilitating entrance into the system, which again should be designed in a way that opens up for a plurality and multiplicity of opt ins, instead of a ‘negative’ freedom of opt out.

## 9. Preliminary Conclusions

This paper has advocated alternative approaches for identifying and engaging with DDDs living with chronic conditions as a way of generating useful insights for re-designing existing chronic care systems and technologies to be more inclusive and effective in tailoring approaches to the abilities of these citizens in a manner that makes the approaches more inclusive and customizable over time. In essence, this approach starts from a premise that services that engage citizens in their own care must be tailored to the pre-existing skills, capacities, and desires of these people rather than being premised on their ability to over-come barriers to entry, imposed by a requirement for a certain pre-determined level of health or eHealth literacy. The approach recognizes that to be successful there is a need to move activities nearer to the individual and maybe also to include their informal care givers, family, or relatives in the dialogue with the service providers.

It is anticipated that this paper will make contributions to the on-going discussions and attempts to re-invigorate health and eHealth approaches to chronic disease management in a manner that is more inclusive and orientated to the needs, wants, and skills of the most vulnerable and disadvantaged in society as a means to emancipate eHealth-mediated chronic care models from their current trajectory that may further disenfranchise these most vulnerable citizens.

This paper presents a framework that supports the identification of DDDs and methods, tools, and techniques to involve, engage, and prepare citizens to be more interactive co-participants in managing their own conditions. This framework also highlights how overcoming the challenges of working with vulnerable citizens can provide a stimulus to improve the whole of the care delivery system towards more people centeredness for chronic care.
